# Influencing attitudes towards antimicrobial use and resistance in companion animals—the impact on pet owners of a short animation in a randomized controlled trial

**DOI:** 10.1093/jacamr/dlae065

**Published:** 2024-05-06

**Authors:** Emma Wright, Lisbeth Rem Jessen, Alice Tompson, Catherine Rutland, David Singleton, Ian Battersby, Isuru Gajanayake, Margo Mosher, Sharon Pfleger, Toby Gemmill, Tim Sparks, Tina M Sørensen, Fergus Allerton

**Affiliations:** The Royal Veterinary College, Hawkshead Lane, Hatfield, Hertfordshire, AL9 7TA, UK; Department of Veterinary Clinical Sciences, University of Copenhagen, Dyrlægevej 16, Frederiksberg C, 1870, Denmark; Department of Public Health, Environments and Society, London School of Hygiene and Tropical Medicine, Keppel Street, London, WC1E 7HT, UK; Simplyhealth, Hambledon House, Waterloo Court, Andover, Hampshire, SP10 1LQ, UK; Institute of Infection, Veterinary and Ecological Sciences, University of Liverpool, Liverpool, UK; Mars Veterinary Health, 18101 SE 6th Way, Vancouver, WA, 98683, USA; Willows Veterinary Centre and Referral Service part of Linnaeus Veterinary Limited, Highlands Road, Shirley, Solihull, B90 4NH, UK; Mars Veterinary Health, 18101 SE 6th Way, Vancouver, WA, 98683, USA; NHS Highland, Public Health Directorate, Larch House, Stoneyfield Business Park, Inverness, IV2 7PA, UK; Willows Veterinary Centre and Referral Service part of Linnaeus Veterinary Limited, Highlands Road, Shirley, Solihull, B90 4NH, UK; Waltham Petcare Science Institute, Freeby Lane, Waltham on the Wolds, Melton Mowbray, Leicestershire, LE14 4RT, UK; Department of Veterinary Clinical Sciences, University of Copenhagen, Dyrlægevej 16, Frederiksberg C, 1870, Denmark; Willows Veterinary Centre and Referral Service part of Linnaeus Veterinary Limited, Highlands Road, Shirley, Solihull, B90 4NH, UK

## Abstract

**Objectives:**

Antimicrobial resistance (AMR) is a vital One Health issue; the rational use of antimicrobials is essential to preserve their efficacy. Veterinarians cite pressure from pet owners as a contributor to antimicrobial prescription. Engaging pet owners in antimicrobial stewardship could reduce this pressure. A short educational animation could facilitate communication of this message. The impact of the animation on participant’s opinions relating to antimicrobial prescribing and awareness of AMR was assessed via a randomized controlled trial.

**Methods:**

A survey was created based on the health belief model. Owners attending six UK veterinary centres were randomized to the intervention or control group (ratio 1:1). All owners completed an agreement level survey of two questions followed by 18 statements scored using a Likert agreement scale. The control group responded without interruption, whereas the animation group was shown the animation after answering the first two questions and five statements.

**Results:**

In total, 647 owners participated in the study; 350 complete responses were analysed. Responses to 10 of 13 statements asked after the animation were significantly different (all *P* < 0.050) between groups, whereas there was no significant difference between groups in response to any of the statements asked before the animation. The animation group were more likely to agree that lower antimicrobial use would help maintain future efficacy (*P* < 0.001) and that requesting antimicrobials from their vet could increase unnecessary use (*P* < 0.001). The animation group were more likely to disagree that they would expect antimicrobials if their pet had diarrhoea (*P* = 0.048).

**Conclusions:**

Pet owners that watched a short AMR engagement animation displayed greater awareness of the impact of AMR and were more likely to support measures in line with antimicrobial stewardship. This behavioural-nudge resource could support owners towards contributing to a multi-faceted approach to AMR.

## Introduction

Antimicrobial resistance (AMR) is a prominent global public health threat that is already contributing to the deaths of millions of individuals worldwide.^1^ Overuse, inappropriate prescribing and inappropriate disposal of antimicrobials risks accelerating the development of drug-resistant pathogens in the environment, and in both humans and animals.^2^

The veterinary profession has a responsibility to ensure prudent use of antimicrobials to help preserve their efficacy and contain AMR.^3^ Antimicrobial stewardship can be considered as strategies or actions promoting responsible antimicrobial use.^4^ In recent years, there have been multiple national and international guidelines developed in companion animal medicine promoting the rational use of antimicrobials.^5,6^ Antimicrobial stewardship initiatives within veterinary medicine advocate withholding prescription of antimicrobials for conditions where such medication would be anticipated to have minimal impact on patient outcome, e.g. acute diarrhoea, vomiting, feline lower urinary tract disease and an upper respiratory tract cough.^6^ These guidelines have been widely disseminated but the target audience for stewardship measures has predominantly been veterinary surgeons.^7–9^ As such, recommendations in veterinary literature have focused on guiding and supporting prescription decision making.^3,10,11^

In human medicine, intervention efforts seeking to improve the knowledge and attitudes of individual prescribers alone often do not address the wider drivers of antimicrobial use, such as the broader economic context, and may not deliver a sustained impact.^12,13^

Antimicrobial prescribing is a highly complex process; a recent review identified 30 factors that influence prescribing behaviour, with patient influence considered among the most important by primary care professionals.^14^ At least some antimicrobial prescription is driven by patient demand. Medical practitioners have indicated a susceptibility to yield to patient pressure, revising their decisions away from their original clinical assessment.^15^ This attitude is echoed in the veterinary profession with veterinarians reporting perceived pressure and direct demands from clients for prescription of antimicrobials.^8,16,17^

In recognition of the key-stakeholder role of patients (and by extrapolation pet owners) in prescribing decisions, stewardship measures should look to directly engage patients and encourage their consideration of the wider AMR implications.^18,19^ However, the most effective ways of interacting and educating the public to help prevent unnecessary antimicrobial use have yet to be established.^20^

Studies have investigated the impact of video animations to reduce patient requests for antimicrobials and to raise awareness of responsible antimicrobial use in human healthcare.^21,22^ However, the role of video animations to educate veterinary clients about AMR has not previously been evaluated.

The aim of this study was to develop and evaluate a short, animated educational film for pet owners.

The primary objective was to determine whether a short educational animation on antimicrobial stewardship could affect pet owner’s opinions on antimicrobial prescribing and their awareness of the importance of AMR. The secondary objective was to evaluate whether participant’s self-defined knowledge of AMR, influenced their receptiveness to the animation.

## Materials and methods

The study was designed in accordance with the CONSORT guidelines as a randomized controlled trial. Ethical approval for this study was obtained from the Royal College of Veterinary Surgeons (2021-83-Wright). The CONSORT reporting guidelines were used in this study (CONSORT S5, available as Supplementary data at *JAC-AMR* Online).^23^

An animated story board was developed to inform pet owners of the threat posed by AMR and to encourage behaviours consistent with effective antimicrobial stewardship. The animated film was iteratively designed with input from a panel of experts in the fields of human healthcare, antimicrobial stewardship and veterinary medicine.

The animation was centred on the story of a dog presented to a veterinarian for acute onset diarrhoea (Supplementary Animation S1). The dog was described as bright and active with a conserved appetite. The pet owner asked the veterinarian whether antimicrobials were required prompting an explanation of AMR, the implications of unnecessary antimicrobial use and the role of pet owners in antimicrobial stewardship.

Commercially available animation software (Vyond^™^) was used to create the 2-minute animated film employing terminology that is familiar to members of the general public and including simple and entertaining animations.

Eligible participants were any pet owner attending one of six veterinary centres in the UK. Pet owners were invited to participate in the study via a brief information slip (Supplementary Owner invitation S4) shown to them on arrival at the clinic. A QR code was incorporated into this information enabling an automatic URL link. The initial page contained a consent form detailing the objectives of the study (without specifically mentioning AMR), and reassuring owners that their answers would be anonymous and that participation would not affect their pet’s care (Supplementary Consent form S2).

Owners who provided informed consent were then taken to the anonymized survey (Supplementary Questionnaire S3). All pet owners completed an agreement level survey of two questions followed by 18 statements scored using a Likert agreement scale. The control group responded to the two questions and 18 statements without interruption, whereas the animation group was shown the animation after answering the first two questions and five statements.

The first question in the survey established the reason for presentation at the vets with answers categorized as either procedure (e.g. vaccination, routine operation), because the pet was unwell, for a check-up appointment, for none of these reasons or other (free text option). Participants were also able to respond that they would rather not answer. The second question required pet owners to self-report their knowledge of AMR on a sliding scale between 0 (no knowledge at all) and 100 (complete knowledge).

The following 18 statements in the survey were structured on one of the constructs: perceived susceptibility, perceived severity, perceived benefits or barriers, self-efficacy or cues to action. Each of these statements were answered on a five-point Likert scale (1 = strongly disagree to 5 = strongly agree). Constructs were based on the health belief model (HBM) a conceptual framework in health behaviour research to guide and evaluate health promotion and disease prevention programmes.^24^ No personal or demographic data were collected from study participants.

Participants were randomly allocated by an integrated, computerized, random number generator (ratio 1:1) to either the animation group or the control group (no animation shown). The randomization was not known to either the participant or study designer until the results were generated. Participants self-enrolled in the study if they elected to follow the QR code provided on a brief information slip. The QR code for each centre was individualized to automatically identify the centre it was submitted from.

Blinding was not necessary as no investigators were present when participants were completing the survey. The study recruitment period was set as a 6-week interval between 10 January 2022 and 21 February 2022.

## Statistical analysis

Data were collected and exported onto Microsoft Excel. For ease of visual comparison, Likert scales were reversed if necessary to ensure that all high values were associated with optimal antimicrobial stewardship. This meant that for all statements the score of 5 reflected the most favourable answer from an antimicrobial stewardship perspective. The reversed scores applied to statements 4, 5, 8, 10, 14, 19 and 20.

Associations between the proportions shown the animation and (i) centre type (referral and primary care) and (ii) reason for visit were tested using chi-square contingency tables.

Owner responses were compared between animation and control groups (and between centre types) using the Mann–Whitney test adjusted for ties. Readers should be aware that, with a large number of tests, some significant results may have arisen by chance alone.

Means as well as medians are presented for groups as although the median is appropriate for categorical data there is a low discriminating ability of this in comparison to the mean.

Perceived knowledge of AMR was evaluated between animation and control groups as well as the relationship between knowledge and mean overall response to statements. This was performed with Spearman rank correlation, and with rank regression using Rfit in R. Rank regression was repeated after excluding answers to question 2 (self-declaration of AMR knowledge) that were unchanged from the default answer of 50. The statements after the animation were separated into their relevant HBM construct. The mean score for each HBM construct was calculated by adding the scores for all statements of the construct type and dividing by the number of statements. Constructs were then compared between the control and animation groups with the Mann–Whitney test adjusted for ties.

There were some HBM constructs represented in both statements before and after the animation was shown. The mean of each HBM construct for statements after the animation was calculated as above and the score of the question asked before the animation was subtracted from this to give a ‘change’ value. Changes were then compared between the control and animation groups with a Mann–Whitney test adjusted for ties.

Statistical significance was taken as *P* < 0.050. Data were analysed using commercially available statistical software [Minitab 21 (Minitab LLC, USA) and Excel (Microsoft USA)] and with R v.4.2.2.

## Results

**Figure 1.**
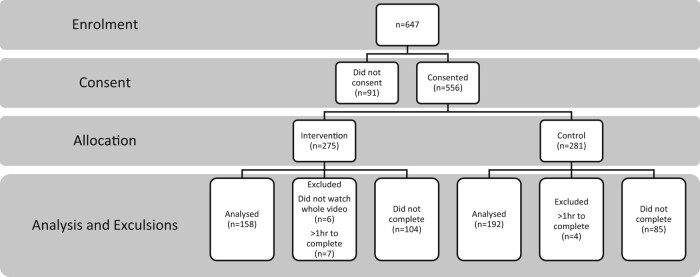
Participant flow diagram showing for each group; the numbers of participants who were randomly assigned, losses and exclusions after randomization and the numbers analysed.

Six hundred and forty-seven (647) pet owners participated in the study. Responses were excluded if they did not proceed past consent (*n* = 91), took longer than 1 hour to complete the survey (*n* = 11), did not watch the entire video (*n* = 6) or did not complete the survey (*n* = 189). There were 350 complete responses and only these are used in subsequent summaries (Figure 1).

Of the six centres involved in the study, the three primary care practices contributed 11.7% of responses whereas 88.3% of responses were from the three practices offering both referral and primary care service. Numbers of responses per centre ranged from 6 to 280 with a median of 13.5.

Ninety-five (27.1%) participants attended the veterinary practice for a check-up, 102 (29.1%) for a procedure and 148 (42.3%) as their pet was unwell. Of 64 cases initially classified as other, 59 could be re-categorized by the authors into the pre-specified categories on the basis of the owner’s description, leaving five (1.4%) visits unclassified.

The animation was viewed by 158 (45.1%) of the 350 participants. The proportion shown the animation did not differ significantly for participants attending primary care (41.5%) or referral/mixed (45.6%) practice (*P* = 0.614). There was no significant difference in responses to statements 2–20 based on centre type.

**Figure 2.**
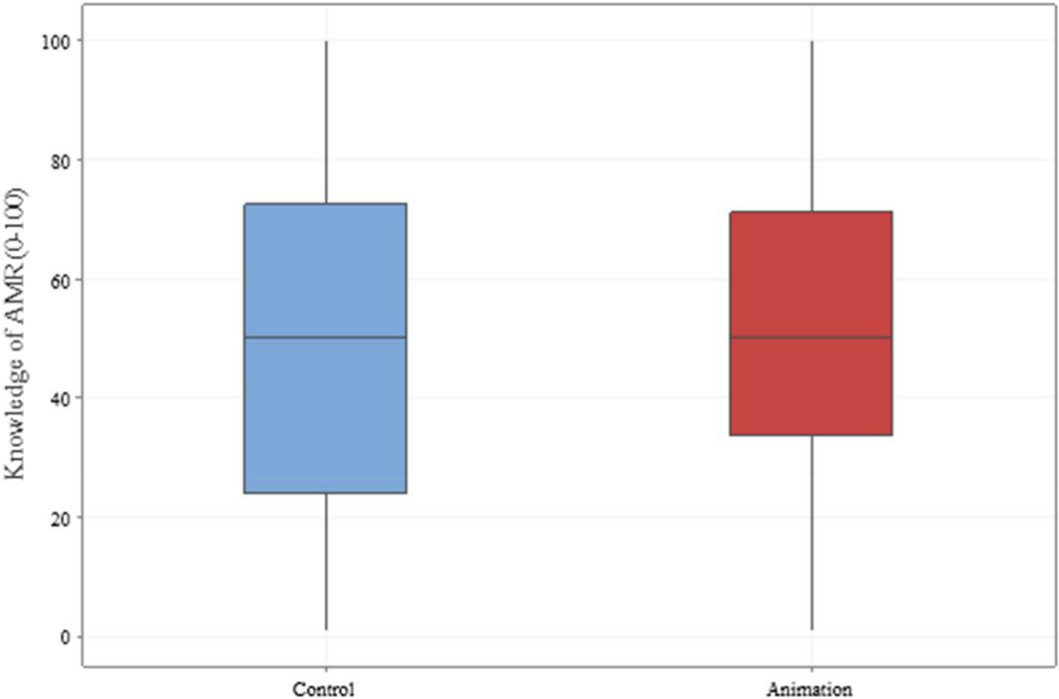
Box and whisker plot of responses for Statement 2. The participants’ self-declared knowledge of AMR was answered on a sliding scale from 0 to 100. The median and interquartile range of responses are demonstrated for the control (blue) and the animation (red) group.

There was no significant association between the proportion of respondents shown the animation and the reason for their visit (*P* = 0.385). Figure 2 shows the distribution of participants’ self-declared knowledge of AMR according to intervention group. No significant difference between groups was detected (*P* = 0.139).

**Figure 3.**
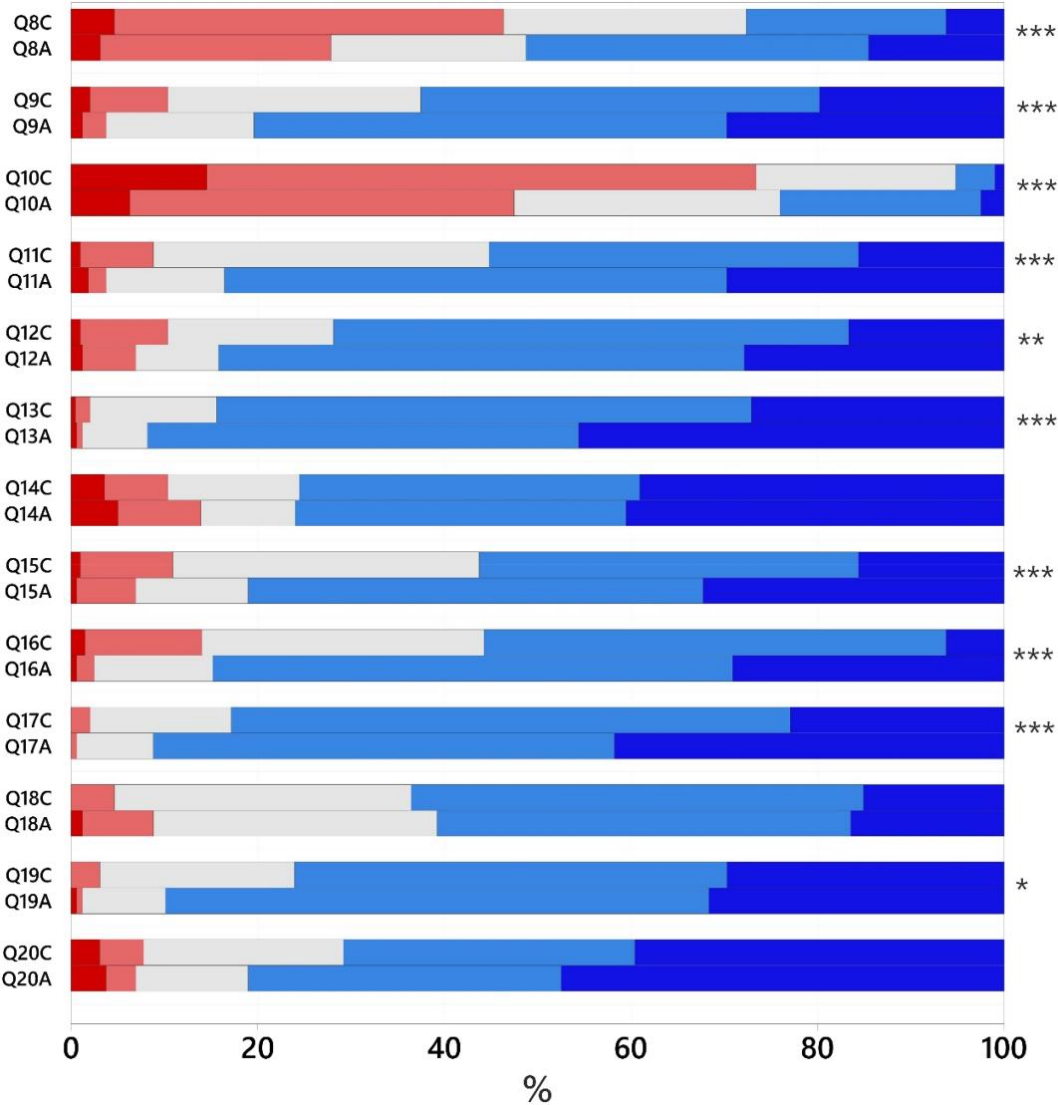
The distribution of responses (%) from strongly disagree (red) to strongly agree (blue) for the control (C) and animation groups (A) for statements 8 to 20. Significance between groups is indicated as follows: *P < 0.05; **0.001 < *P* < 0.01; ***P < 0.001.

There were no significant differences between the control and animation groups in response to statements 3–7, asked before the animation (Table 1). Of the 13 statements asked after the animation (statements 8–20), responses to 10/13 statements were significantly different between the animation and control groups (Table 2). The distribution of scores for statements 8–20 for both groups is illustrated in Figure 3. The scores for statements of each HBM construct were combined and the significance assessed, these values are demonstrated in Table 3.

**Table 1.** Responses of the control and animation group to statements 3 to 7 (asked before the animation was presented) indicating the median and mean scores, confidence interval, the test statistic (*W*) of the Mann–Whitney test and *P* value

Statement	Group	Median (mean)	95% confidence interval for median (for mean)	*W*	*P*
3. I have received antibiotics for my pet when I was not sure they were needed	Control	2 (2.00)	2–2 (1.87–2.13)	33 320	0.672
	Animation	2 (2.03)	2–2 (1.89–2.16)		
4. In the past I have felt my pet wasn’t going to be prescribed antibiotics, so I asked for them	Control	4 (4.25)	4–5 (4.12–4.38)	34 913	0.164
	Animation	4 (4.11)	4–5 (3.96–4.26)		
5. I would rather use antibiotics just in case my pet has an infection than risk them becoming more ill	Control	3 (3.04)	3–3 (2.87–3.21)	33 182	0.574
	Animation	3 (3.11)	3–3.8 (2.91–3.20)		
6. Many people could die or suffer if bacteria become ever more resistant to antibiotics	Control	4 (4.18)	4–4 (4.07–4.29)	34 858	0.182
	Animation	4 (4.06)	4–4 (3.93–4.19)		
7. I would tell my vet if I did not think antibiotics were necessary for my pet	Control	3 (3.27)	3–4 (3.12–3.42)	32 774	0.305
	Animation	4 (3.39)	3–4 (3.23–3.54)		

Likert scale responses; 1, Strongly Disagree; 2, Disagree; 3, Neutral; 4, Agree; 5, Strongly Agree. Likert scales for statement 4 and 5 were reversed to ensure that all high values were associated with optimal antimicrobial stewardship.

**Table 2.** Responses of the control and animation group to statements 8 to 20 indicating the median and mean scores, confidence interval, the test statistic (*W*) of the Mann–Whitney test and *P* value

Statement	Group	Median (mean)	95% confidence interval for median (for mean)	*W*	*P*
8. I will ask my vet for antibiotics if I feel my pet needs them (Reversed: 1, Strongly Agree; 5, Strongly Disagree)	Control	3 (2.83)	2–3 (2.68–2.97)	29 640	<0.001
	Animation	4 (3.35)	3–4 (3.18–3.52)		
9. Antibiotic resistance could impact me personally	Control	4 (3.70)	4–4 (3.56–3.83)	30 491	<0.001
	Animation	4 (4.05)	4–4 (3.92–4.18)		
10. Delaying a prescription for an antibiotic could risk my pet getting worse (Reversed: 1, Strongly Agree; 5, Strongly Disagree)	Control	2 (2.18)	2–2 (2.07–2.29)	28 876	<0.001
	Animation	3 (2.73)	2–3 (2.58–2.88)		
11. In the future there may be no effective antibiotic available to treat my pet if they have an infection	Control	4 (3.61)	3–4 (3.48–3.73)	28 943	<0.001
	Animation	4 (4.08)	4–4 (3.95–4.20)		
12. Requesting antibiotics from my vet may increase unnecessary use	Control	4 (3.77)	4–4 (3.65–3.90)	30 978	0.001
	Animation	4 (4.04)	4–4 (3.91–4.17)		
13. Antibiotic resistance could impact my pet	Control	4 (4.09)	4–4 (3.99–4.19)	30 517	<0.001
	Animation	4 (4.35)	4–5 (4.24–4.46)		
14. As a pet owner I have no role to play in using antibiotics wisely (Reversed: 1, Strongly Agree; 5, Strongly Disagree)	Control	4 (4.01)	4–4 (3.85–4.16)	33 631	0.942
	Animation	4 (3.97)	4–4 (3.79–4.16)		
15. All antibiotic use can increase the risk of bacteria becoming resistant	Control	4 (3.60)	3–4 (3.47–3.73)	29 252	<0.001
	Animation	4 (4.06)	4–4 (3.92–4.19)		
16. I would be happy to wait a few days to see if my pet got better without antibiotics	Control	4 (3.46)	3–4 (3.34–3.58)	27 481	<0.001
	Animation	4 (4.11)	4–4 (3.99–4.22)		
17. Using fewer antibiotics will help keep them working in the future.	Control	4 (4.04)	4–4 (3.94–4.13)	30 342	<0.001
	Animation	4 (4.32)	4–4 (4.22–4.43)		
18. I would like to talk to my vet about the risks and benefits of antibiotic treatment for my pet	Control	4 (3.74)	4–4 (3.63–3.85)	34 144	0.609
	Animation	4 (3.67)	4–4 (3.53–3.81)		
19. If my pet has diarrhoea, I would expect them to be given antibiotics (Reversed: 1, Strongly Agree; 5, Strongly Disagree)	Control	4 (4.03)	4–4 (3.91–4.14)	31 996	0.048
	Animation	4 (4.20)	4–4 (4.09–4.30)		
20. Antibiotic resistance poses little threat to animal and human health (Reversed: 1, Strongly Agree; 5, Strongly Disagree)	Control	4 (3.99)	4–4 (3.85–4.14)	32 011	0.057
	Animation	4 (4.18)	4–5 (4.02–4.33)		

Likert scales for statements 8, 10, 14, 19 and 20 were reversed to ensure that all high values were associated with optimal antimicrobial stewardship.

**Table 3.** Combined HBM construct scores for the statements after the animation for the control and animation groups indicating the median and mean scores, confidence interval, the test statistic (*W*) of the Mann–Whitney test and *P* value

HBM construct	Associated statements	Group	Median (mean)	95% confidence interval for median (for mean)	*W*	*P*
Perceived susceptibility	9, 13, 15	Control	3.83 (3.80)	3.67–4.00 (3.70–3.89)	28 810	<0.001
		Animation	4.00 (4.15)	4.00–4.33 (4.05–4.25)		
Perceived seriousness	11, 20	Control	4.00 (3.80)	3.50–4.00 (3.69–3.92)	30 168	<0.001
		Animation	4.00 (4.13)	4.00–4.50 (4.01–4.24)		
Perceived benefits and barriers	10, 12, 17	Control	3.33 (3.33)	3.33–3.33 (3.25–3.41)	28 095	<0.001
		Animation	3.67 (3.70)	3.67–3.67 (3.60–3.79)		
Self-efficacy	14, 16	Control	4.00 (3.73)	3.50–4.00 (3.63–3.84)	30 234	<0.001
		Animation	4.00 (4.04)	4.00–4.00 (3.93–4.15)		
Cues to Action	8, 18, 19	Control	3.33 (3.53)	3.33–3.67 (3.45–3.61)	30 373	<0.001
		Animation	3.67 (3.74)	3.67–4.00 (3.65–3.83)		

Three HBM constructs were represented in statements shown both before (statements 3–7) and after the animation (statements 8–20). The change values (i.e. the difference in mean score for constructs included in statements 3–7 versus 8–20) were significantly different (*P* < 0.050) for the constructs of perceived seriousness and perceived benefits and barriers between the control and animation groups (Table 4). The change was not significant for the construct self-efficacy (*P* = 0.163).

**Table 4.** HBM constructs represented both before and after the animation

HBM construct	Associated statements: after	Associated statements: before	Group	Median (mean)	95% confidence interval for median (for mean)	*W*	*P*
Perceived seriousness	11, 20	6	Control	−0.50 (−0.38)	−0.50 to 0.00 (−0.48 to −0.28)	28 476	<0.001
			Animation	0.00 (0.06)	0.00–0.00 (−0.06 to 0.20)		
Perceived benefits and barriers	10, 12, 17	5	Control	0.33 (0.29)	0.00–0.33 (0.14–0.43)	31 459	0.017
			Animation	0.67 (0.59)	0.33–0.67 (0.42–0.76)		
Self-efficacy	14, 16	7	Control	0.50 (0.46)	0.00–0.50 (0.31–0.61)	32 399	0.163
			Animation	0.50 (0.66)	0.50–1.00 (0.48–0.83)		

The ‘change’ value of the average combined score for statements after the animation minus the score of the statement before. For the control and animation groups are given the median and mean ‘change’, confidence interval, the test statistic (*W*) of the Mann–Whitney test and *P* value.

**Figure 4.**
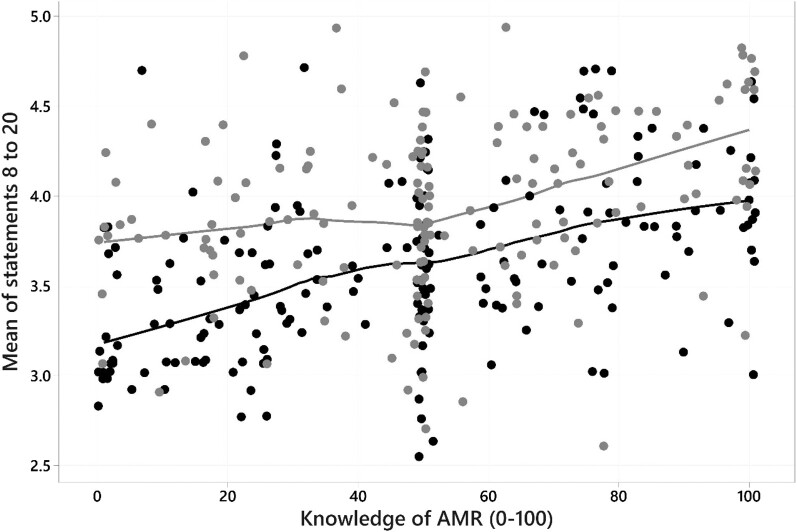
Scatter graph showing relationship between statement 2 and mean of statements 8 to 20 with lowess smoothed lines for control (dark grey) and animation (light grey) groups.

A Spearman rank correlation coefficient found a positive correlation (*r*_s _= 0.402, *P* < 0.001) between self-declared AMR knowledge and mean score (i.e. the greater the participant self-declared knowledge score on statement 2, the more likely they were to favour statements indicating improved antimicrobial stewardship). A rank regression undertaken using Rfit in R of mean score on declared AMR knowledge, group and their interaction confirmed a significant positive relationship with AMR knowledge (*P* < 0.001), a significant difference between groups (estimated at 0.41, *P* < 0.001) but no significant interaction (*P* = 0.224). Conclusions were unchanged when the large number (*n* = 77, 22%) of knowledge responses at exactly 50 were excluded. Figure 4 shows lowess smoothed lines for the animation and control groups according to their response to statement 2. The near-parallel nature of these lines, and the results from rank regression described previously, indicate that the impact of the animation was similar across all participants independent of their self-declared knowledge of AMR.

The median of the control group and animation group standard deviations for statements 8 to 20 was 0.86. *Post hoc* sample size verification was performed based on a two-tailed two-sample *t*-test but with a 15% uplift in sample size for the equivalent nonparametric test (Mann–Whitney). Assuming a power of 0.80, a significance of 0.050 and an effect size based on means of 0.3 results in a sample size per group of 130, uplifted to 150 per group.

## Discussion

The animation, designed in this study, had a positive influence on the attitudes of pet owners towards antimicrobial stewardship. Although the changes in statement scores were generally small, responses to 10 of 13 statements were consistent with a greater stewardship sentiment for the intervention group. These findings suggest that this simple resource could facilitate communication of an important educational message to pet owners. By increasing owner awareness and appreciation of the dangers associated with AMR, veterinarians will be better placed to engage in meaningful conversations around antimicrobial use and ultimately to avoid unnecessary prescription.

Perceived pressures from pet owners have been found to influence veterinarian decision making around antimicrobial prescription in several studies. A qualitative study that considered the perspectives of both pet owners and veterinarians found that pet owners felt that veterinarians were responsible for ‘overuse’.^17^ Meanwhile the veterinarians felt the owners applied pressure for antimicrobials, although this was often inferred rather than explicitly demanded by owners.^17^ In a survey of pet owners in Australia, 15% of pet owners reported explicitly requesting antimicrobials from their vet although the appropriateness of requests was not assessed.^25^ In addition to this, an overall poor public understanding of AMR has been highlighted,^26^ including a lack of awareness of the causes of AMR.^27^ Inadequate pet owner comprehension of AMR and its drivers has the potential to restrict stewardship efforts.^17^ This study (and the resource created herein) seeks to address some of those deficits.

The pet owner is a decision-making surrogate for the patient, thus owner expectations and owner pressure are key factors when aiming to diminish requests for antimicrobial prescription.^28,29^ By raising the topic of AMR in the waiting room, this animation has the potential to rapidly introduce key concepts and ultimately to bolster the stewardship message prior to contact with the prescriber.

Given the time pressures inherent to veterinary consultations, discussion of AMR is inevitably overlooked on occasion.^30^ Preloading pet owners with some information could take some of the responsibility away from the consultation. Educational animations have been trialled in human medical outpatient settings with variable impact on knowledge, recollection of stewardship messages and intention to request antimicrobials.^21,22^

Importantly the constructs of the HBM were used in this study to encompass all aspects of health behaviour,^31^ and offered a quantifiable means to evaluate the impact of the animation on pet owner attitudes. Interventions founded on psychology and health behaviour change principles have been shown successful in prompting behaviour change.^32^ The HBM model has previously been used in veterinary research assessing owner compliance with diet recommendations,^33^ demonstrating the adaptability of this tool to veterinary medicine. This study represents the first time the HBM has been applied to antibiotic use in the companion animal setting.

In this study, some HBM constructs were incorporated into the survey both before and after the animation. This enabled comparison of the change values (the differences between an individual’s responses to grouped statements) for several constructs between the animation and control group. The finding of statistically significant differences between the control and animation groups for both perceived severity and benefits and barriers suggests that the animation effectively communicated the seriousness of the threat from AMR and an awareness of the values of positive action to reduce this risk. The change was not statistically significant for the statements representing self-efficacy suggesting that pet owners may still not value their own role to affect the outcome. Similar findings have been described in the medical field where patients have high levels of trust in healthcare practitioners and therefore assume a passive role in decision making.^18^ Potentially, pet owners believe that veterinarians prescribe antimicrobials only when appropriate, and thus consider their role (self-efficacy) in antimicrobial stewardship to be limited. Of all the HBM constructs, self-efficacy is the least researched and developed element of the model. Consequently, it is sometimes excluded from studies that only include the core concepts discussed previously. However, the concept of self-efficacy is significant in the context of fear based public health campaigns to empower observers to carry out recommended behaviours hence its inclusion in this study.^34^

In veterinary medicine, canine acute diarrhoea has a reported antimicrobial prescription rate of up to 70%,^35–39^ although systemically administered (oral or injectable) antimicrobials are considered unwarranted in most such patients.^10,40–42^ Consequently, the animation was centred on canine acute diarrhoea but the message was sufficiently broad to be applicable to other clinical scenarios. By, reducing owner expectations of receiving antimicrobials, it was hoped the animation would reduce veterinarian perceived pressure from pet owners.^43^ Similar to other behaviour change interventions, the animation was designed to discourage requests for antimicrobials by emphasizing the threat and consequences of AMR.^32,44^ Scores for pet owners in the animation group indicated that they would be less likely to expect or request antimicrobials if their pet had diarrhoea (statements 8 and 19), would be prepared to wait longer before considering antimicrobial use (statement 16) and would also be more likely to consider delayed prescription of antimicrobials (statement 10). There was also a demonstration of greater awareness that requesting antimicrobials could increase unnecessary use (statement 12). The animation group also demonstrated a greater awareness of the importance and impact of AMR. Direct references to the consequences of AMR, including both animal and human health concerns, were incorporated into the animation and may have influenced a perception of personal susceptibility. If AMR is perceived as likely to affect an individual rather than representing a more nebulous public health threat, the probability of engagement in stewardship activities will increase.^18^ Reminding owners that antimicrobial use in their pets could affect their own health may represent a more potent motivator for behavioural change than generic threats to the wider animal community.

Overall, there was a small but significant difference to all the HBM constructs assessed after the animation. This behavioural nudge could influence owners sufficiently towards becoming a positive actor in a multi-faceted approach to antimicrobial stewardship.

The control and animation groups displayed similar scores for self-defined AMR knowledge and in responses to all the statements shown before the animation. Given this homogeneity, any inter-group difference in the responses to the statements after the animation were considered attributable to the impact of the animation. It was anticipated that owners with a greater base knowledge of AMR (albeit self-professed) would respond more favourably (from a stewardship perspective) to all statements. Indeed, there was a positive correlation between higher self-declared knowledge of AMR and the likelihood of favouring statements in line with improved stewardship. Nonetheless, the impact of the animation was present for all participants independent of previous knowledge, indicating that the animation was effective and appropriate for a wide spectrum of the pet-owning public.

There were several limitations to this study. No data were collected regarding the characteristics of owners participating in the study, hence there may be some degree of bias impacting the generalizability of the findings. The longer-term impact on pet owner behaviour changes or antimicrobial prescribing rates were not evaluated. In one study, the documented immediate reduction in the intention to request antimicrobials was not sustained on a 6-week follow-up questionnaire.^22^ However, the resource was designed with the intention of being shown in the waiting room, just before consultation. Hopefully, with further reinforcement from a similar message from the veterinarian, the desired impact on antimicrobial prescription rates can be achieved. As with many public health announcements, frequent exposure is likely to be necessary to create sustained behavioural modification. The study was intention-based and as such one limitation is that it is unknown how the respondents would have acted in the consultation. Self-defined AMR knowledge responses could potentially have been influenced as the scale provided for responses was preset at 50%. Respondents may have been reluctant to move the dial too far leading to the observed clustering around the middle value. There was a bias towards clients attending one mixed first opinion/referral practice. It is acknowledged that although there was no significant difference between responses from referral or primary care practices, the responses may not be representative of the general pet-owning public.

### Conclusion

AMR is a prominent global public health threat.^1^ Only through a collaborative and comprehensive approach involving all relevant stakeholders, including antimicrobial use guideline developers, veterinarians, pet owners, representatives of the pharmaceutical industry, public health campaigns and diagnostic laboratories, can meaningful change in antimicrobial use be achieved.^45^

The animation designed and tested in this study has found a small but important positive impact on pet owners’ attitudes towards antimicrobial stewardship. Hence the animation can be used as part of a multi-faceted approach to AMR to effectively change pet owner intentions and behaviours towards antimicrobial use. This will reinforce the link to other AMR messages conveyed across all sectors of healthcare.

## Authorship

Emma WRIGHT—conception, implementation, manuscript writing, review; Lisbeth Rem JESSEN—conception, review; Alice THOMPSON PhD—conception, review; Catherine RUTLAND—conception, review; David SINGLETON—conception, review; Ian BATTERSBY—conception, review; Isuru GAJANAYAKE—conception, review; Margo MOSHER—conception; Sharon PFLEGER—conception, review; Toby GEMMILL—conception, review; Tim SPARKS—conception, manuscript writing, review; Tina M SOERENSEN—conception, review; Fergus ALLERTON—conception, implementation, manuscript writing, review.

## Supplementary Material

dlae065_Supplementary_Data

## Supplementary data

Supplement S1. Animation link; Supplement S2. Consent form; Supplement S3. Questionnaire; Supplement S4. Owner invitation to study and Supplement S5. CONSORT guidelines compliance checklist are available as Supplementary data at *JAC-AMR* Online.
