# Supplementary material for: Influencing attitudes towards antimicrobial use and resistance in companion animals—the impact on pet owners of a short animation in a randomized controlled trial
Source: JAC Antimicrob Resist. 2024 May 6;6(3):dlae065. doi: 10.1093/jacamr/dlae065 (PMC11073752; doi:10.1093/jacamr/dlae065)
Supplement: dlae065_Supplementary_Data [file dlae065_supplementary_data.docx]

**Supplement 1 - Animation Link**

<https://youtu.be/4ApEAfN4dWU>


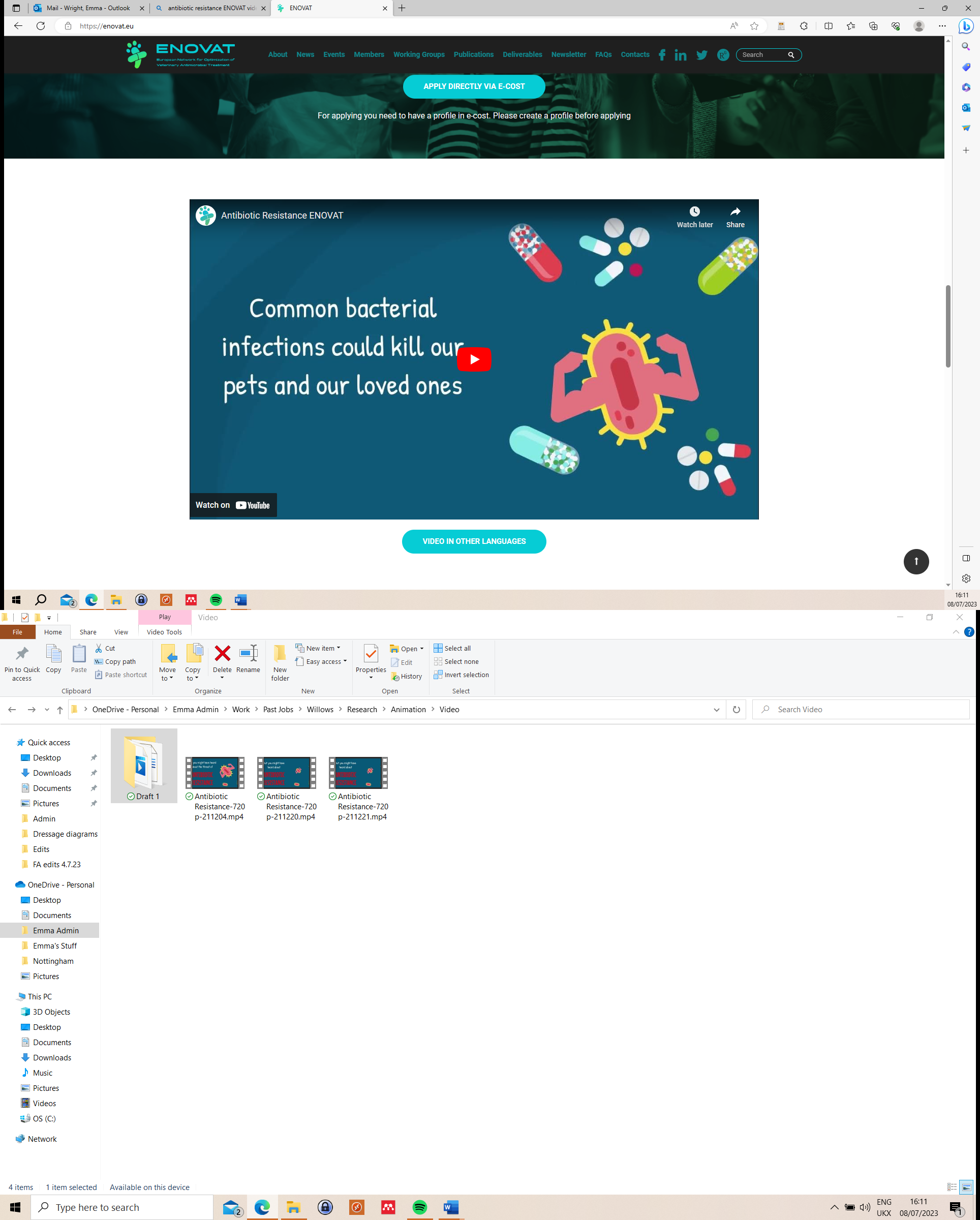


**Supplement 2 - Consent form**

Thank you for your interest in helping with this study. A short survey has been designed to gauge the understanding and opinions of pet owners relating to important aspects of animal health and welfare. Participation should take no longer than 10 minutes.

The study is completely anonymised, so there is no personal data collected. You can stop the survey at any point. There is no obligation to participate in this study and it will have no impact on your pet’s health or the level of care you receive today or in the future.

If you have any further questions, please do not hesitate to contact the primary investigators: [emma.wright2@willows.uk.net](mailto:emma.wright2@willows.uk.net) or fergus.allerton@willows.uk.net

By accessing this link you are consenting to your answers being included in the study. As the study is anonymous it is not possible to remove responses once completed or to contact you later.

**Supplement 3 - Questionnaire**

**Questions asked before video**

1. Why is your pet presenting to the vet today?
   1. Procedure (e.g. vaccination, routine operation)
   2. Because they are unwell
   3. For a check-up appointment
   4. None of these reasons
   5. Would rather not say
   6. other (free text)
2. How much do you know about antibiotic resistance?
   1. On a scale of 0-10 (a little – a lot) how much do you know about antibiotic resistance – sliding scale
3. I have received antibiotics for my pet when I was not sure they were needed
4. In the past I have felt my pet wasn't going to be prescribed antibiotics, so I asked for them
5. I would rather use antibiotics just in case my pet has an infection than risk them becoming more ill
6. Many people could die or suffer if bacteria become ever more resistant to antibiotics
7. I would tell my vet if I did not think antibiotics were necessary for my pet

**Questions after video**

1. I will ask my vet for antibiotics if I feel my pet needs them
2. Antibiotic resistance could impact me personally
3. Delaying a prescription for an antibiotic could risk my pet getting worse
4. In the future there may be no effective antibiotic available to treat my pet if they have an infection
5. Requesting antibiotics from my vet may increase unnecessary use
6. Antibiotic resistance could impact my pet
7. As a pet owner I have no role to play in using antibiotics wisely
8. All antibiotic use can increase the risk of bacteria becoming resistant
9. I would be happy to wait a few days to see if my pet got better without antibiotics
10. Using fewer antibiotics will help keep them working in the future
11. I would like to talk to my vet about the risks and benefits of antibiotic treatment for my pet
12. If my pet has diarrhoea, I would expect them to be given antibiotics
13. Antibiotic resistance poses little threat to animal and human health

**Supplement 4 – Owner invitation to study**

**Please Help!!**

**Pet Owner Survey**

This is an anonymous survey to gain the opinions of pet owners

Please scan the QR code with the camera on your phone


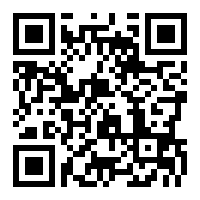
This will direct you to our survey with an initial information page

**Supplement 5 - Reporting checklist for randomised trial based on the CONSORT guidelines.**

|  |  | Reporting Item | Page Number |
| --- | --- | --- | --- |
| **Title and Abstract** |  |  |  |
| Title | [#1a](https://www.goodreports.org/reporting-checklists/consort/info/#1a) | Identification as a randomized trial in the title. | 1 |
| Abstract | [#1b](https://www.goodreports.org/reporting-checklists/consort/info/#1b) | Structured summary of trial design, methods, results, and conclusions | 3 |
| **Introduction** |  |  |  |
| Background and objectives | [#2a](https://www.goodreports.org/reporting-checklists/consort/info/#2a) | Scientific background and explanation of rationale | 4-5 |
| Background and objectives | [#2b](https://www.goodreports.org/reporting-checklists/consort/info/#2b) | Specific objectives or hypothesis | 5 |
| **Methods** |  |  |  |
| Trial design | [#3a](https://www.goodreports.org/reporting-checklists/consort/info/#3a) | Description of trial design (such as parallel, factorial) including allocation ratio. | 5-6 |
| Trial design | [#3b](https://www.goodreports.org/reporting-checklists/consort/info/#3b) | Important changes to methods after trial commencement (such as eligibility criteria), with reasons | n/a |
| Participants | [#4a](https://www.goodreports.org/reporting-checklists/consort/info/#4a) | Eligibility criteria for participants | 5-6 |
| Participants | [#4b](https://www.goodreports.org/reporting-checklists/consort/info/#4b) | Settings and locations where the data were collected | 5-6 |
| Interventions | [#5](https://www.goodreports.org/reporting-checklists/consort/info/#5) | The experimental and control interventions for each group with sufficient details to allow replication, including how and when they were actually administered | 6 |
| Outcomes | [#6a](https://www.goodreports.org/reporting-checklists/consort/info/#6a) | Completely defined prespecified primary and secondary outcome measures, including how and when they were assessed | 6 |
| Outcomes | [#6b](https://www.goodreports.org/reporting-checklists/consort/info/#6b) | Any changes to trial outcomes after the trial commenced, with reasons | n/a |
| Sample size | [#7a](https://www.goodreports.org/reporting-checklists/consort/info/#7a) | How sample size was determined. | 9 |
| Sample size | [#7b](https://www.goodreports.org/reporting-checklists/consort/info/#7b) | When applicable, explanation of any interim analyses and stopping guidelines | n/a |
| Randomization - Sequence generation | [#8a](https://www.goodreports.org/reporting-checklists/consort/info/#8a) | Method used to generate the random allocation sequence. | 6 |
| Randomization - Sequence generation | [#8b](https://www.goodreports.org/reporting-checklists/consort/info/#8b) | Type of randomization; details of any restriction (such as blocking and block size) | 6 |
| Randomization - Allocation concealment mechanism | [#9](https://www.goodreports.org/reporting-checklists/consort/info/#9) | Mechanism used to implement the random allocation sequence (such as sequentially numbered containers), describing any steps taken to conceal the sequence until interventions were assigned | 6 |
| Randomization - Implementation | [#10](https://www.goodreports.org/reporting-checklists/consort/info/#10) | Who generated the allocation sequence, who enrolled participants, and who assigned participants to interventions | 6 |
| Blinding | [#11a](https://www.goodreports.org/reporting-checklists/consort/info/#11a) | If done, who was blinded after assignment to interventions (for example, participants, care providers, those assessing outcomes) and how. | 6 |
| Blinding | [#11b](https://www.goodreports.org/reporting-checklists/consort/info/#11b) | If relevant, description of the similarity of interventions | n/a |
| Statistical methods | [#12a](https://www.goodreports.org/reporting-checklists/consort/info/#12a) | Statistical methods used to compare groups for primary and secondary outcomes | 6-7 |
| Statistical methods | [#12b](https://www.goodreports.org/reporting-checklists/consort/info/#12b) | Methods for additional analyses, such as subgroup analyses and adjusted analyses | 6-7 |
| **Results** |  |  |  |
| Participant flow diagram (strongly recommended) | [#13a](https://www.goodreports.org/reporting-checklists/consort/info/#13a) | For each group, the numbers of participants who were randomly assigned, received intended treatment, and were analysed for the primary outcome | Figure 1 |
| Participant flow | [#13b](https://www.goodreports.org/reporting-checklists/consort/info/#13b) | For each group, losses and exclusions after randomization, together with reason | Figure 1 |
| Recruitment | [#14a](https://www.goodreports.org/reporting-checklists/consort/info/#14a) | Dates defining the periods of recruitment and follow-up | 6 |
| Recruitment | [#14b](https://www.goodreports.org/reporting-checklists/consort/info/#14b) | Why the trial ended or was stopped | 6 |
| Baseline data | [#15](https://www.goodreports.org/reporting-checklists/consort/info/#15) | A table showing baseline demographic and clinical characteristics for each group | n/a |
| Numbers analysed | [#16](https://www.goodreports.org/reporting-checklists/consort/info/#16) | For each group, number of participants (denominator) included in each analysis and whether the analysis was by original assigned groups | 7 |
| Outcomes and estimation | [#17a](https://www.goodreports.org/reporting-checklists/consort/info/#17a) | For each primary and secondary outcome, results for each group, and the estimated effect size and its precision (such as 95% confidence interval) | Table 1 &2 |
| Outcomes and estimation | [#17b](https://www.goodreports.org/reporting-checklists/consort/info/#17b) | For binary outcomes, presentation of both absolute and relative effect sizes is recommended | 7-9 |
| Ancillary analyses | [#18](https://www.goodreports.org/reporting-checklists/consort/info/#18) | Results of any other analyses performed, including subgroup analyses and adjusted analyses, distinguishing pre-specified from exploratory | n/a |
| Harms | [#19](https://www.goodreports.org/reporting-checklists/consort/info/#19) | All important harms or unintended effects in each group (For specific guidance see CONSORT for harms) | n/a |
| **Discussion** |  |  |  |
| Limitations | [#20](https://www.goodreports.org/reporting-checklists/consort/info/#20) | Trial limitations, addressing sources of potential bias, imprecision, and, if relevant, multiplicity of analyses | 11 |
| Generalisability | [#21](https://www.goodreports.org/reporting-checklists/consort/info/#21) | Generalisability (external validity, applicability) of the trial findings | n/a |
| Interpretation | [#22](https://www.goodreports.org/reporting-checklists/consort/info/#22) | Interpretation consistent with results, balancing benefits and harms, and considering other relevant evidence | 9-12 |
| Registration | [#23](https://www.goodreports.org/reporting-checklists/consort/info/#23) | Registration number and name of trial registry | n/a |
| **Other information** |  |  |  |
| Interpretation | [#22](https://www.goodreports.org/reporting-checklists/consort/info/#22) | Interpretation consistent with results, balancing benefits and harms, and considering other relevant evidence | 9-12 |
| Registration | [#23](https://www.goodreports.org/reporting-checklists/consort/info/#23) | Registration number and name of trial registry | n/a |
| Protocol | [#24](https://www.goodreports.org/reporting-checklists/consort/info/#24) | Where the full trial protocol can be accessed, if available | n/a |
| Funding | [#25](https://www.goodreports.org/reporting-checklists/consort/info/#25) | Sources of funding and other support (such as supply of drugs), role of funders | 13 |

None The CONSORT checklist is distributed under the terms of the Creative Commons Attribution License CC-BY. This checklist can be completed online using <https://www.goodreports.org/>, a tool made by the [EQUATOR Network](https://www.equator-network.org) in collaboration with [Penelope.ai](https://www.penelope.ai)
